# Altered Resting-State Functional Activity in Medication-Naive Patients With First-Episode Major Depression Disorder vs. Healthy Control: A Quantitative Meta-Analysis

**DOI:** 10.3389/fnbeh.2019.00089

**Published:** 2019-05-07

**Authors:** Xiaoyue Ma, Jia Liu, Taiyuan Liu, Lun Ma, Wenhui Wang, Shaojie Shi, Yan Wang, Qiyong Gong, Meiyun Wang

**Affiliations:** ^1^Department of Radiology, The First Affiliated Hospital of Zhengzhou University, Zhengzhou, China; ^2^Academy of Medical Sciences, Zhengzhou University, Zhengzhou, China; ^3^Department of Radiology, Zhengzhou University People's Hospital and Henan Provincial People's Hospital, Zhengzhou, China; ^4^Henan Key Laboratory of Neurological Imaging, Zhengzhou, China; ^5^Department of Radiology, Union Hospital of Tongji Medical College, Huazhong University of Science and Technology, Wuhan, China; ^6^Medical School, Henan University, Zhengzhou, China; ^7^Department of Radiology, West China Hospital of Sichuan University, Huaxi MR Research Center (HMRRC), Chengdu, China; ^8^Henan Provincial Clinical Big Data Analysis and Service Engineering Research Center, Zhengzhou, China

**Keywords:** major depressive disorder, resting-state functional activity, voxel-based neuroimaging, seed-based d mapping, meta-analysis

## Abstract

**Background:** There is an urgent need for a meta-analysis that characterizes the brain states of major depression disorder (MDD) patients and potentially provides reliable biomarkers, because heterogeneity in the results of resting-state functional neuroimaging has been observed between studies, with some patients not showing the consistent changes, or even opposite patterns. Thus, we evaluated consistent regional brain activity alterations in medication-naive patients with first-episode unipolar MDD and compared the results with those in healthy controls (HCs).

**Methods:** A systematic database search was conducted (in PubMed, Ovid, and Web of Knowledge) between January 1984 and July 2016 to select resting-state functional activity studies with a voxel-wise analysis in MDD. We used anisotropic effect size-signed differential mapping to perform a whole-brain meta-analysis, comparing functional alterations between first-episode medication-naive unipolar MDD patients and HCs by integrating the studies. In addition, subgroup meta-analysis was conducted to control for the MRI analysis method. Moreover, the meta-regression analyses were performed to examine the potential effects of mean age, education duration, illness duration, and severity of depressive symptoms.

**Results:** A total of 12 studies were included, comparing 313 MDD patients with 283 HCs. The pooled and subgroup meta-analysis found that the MDD patients showed hyperactivity in the left parahippocampal gyrus, left supplementary motor area, left amygdala, left hippocampus, and left middle frontal gyrus (MFG; orbital part), and hypoactivity in the left lingual gyrus, left middle occipital gyrus, right cuneus cortex, right MFG (orbital part), and left cerebellum. In the meta-regression analyses, the mean illness duration was positively associated with hyper-activation in the left parahippocampal gyrus and hypoactivation in the hemispheric lobule IV/V of the left cerebellum.

**Conclusions:** This meta-analysis indicated that MDD patients had significant and robust resting-state brain activity alteration in amygdala, left hippocampus and other regions, which implicated this finding in the pathophysiology of cognitive and emotional impairment in MDD patients.

## Introduction

Depressive disorders constitute a common group of psychiatric disorders with high prevalence (Rosenstrom and Jokela, 2017). With the most conservative estimate of 350 million patients with depression, depression is one of the leading causes of disabilities worldwide (Caan, 2015); however, its clinical definition remains a debated topic. Recent studies have investigated depressive disorder at the level of individual symptoms that define major depressive disorder (MDD) (Fried and Nesse, 2015). The current MDD diagnosis depends on subjective symptoms, experiences and perceptions, requiring the presence of at least one of the two core symptoms: (1) depressed mood and/or (2) markedly diminished interest or pleasure in all, or almost all, activities (Rosenstrom and Jokela, 2017). Reliable biomarkers that can improve the sensitivity and specificity estimates of diagnostic and treatment strategies for MDD are lacking. Scientists have proposed that neuroimaging has the “diagnostic potential” of finding anomalies in brain structure, function, and neurochemistry in patients with depression (Kambeitz et al., 2016).

With the advances in the development of modern imaging techniques in the last few decades, multiple neuroimaging modalities, such as functional magnetic resonance imaging (fMRI), single-photon emission computed tomography (SPECT), and positron emission tomography (PET), have greatly increased the current understanding of altered brain activity in MDD. In general, there are two analysis methods to quantify fMRI resting-state activity: amplitude of low-frequency fluctuation (ALFF) and regional homogeneity (ReHo). In addition, an improved ALFF method, fractional ALFF (fALFF), could improve the sensitivity and specificity in examining regional spontaneous fun (Liu et al., 2013). Moreover, both regional cerebral blood flow (rCBF) and glucose metabolism (rCMglu), produced by arterial spin labeling (ASL)/PET/SPECT, could be used to detect altered brain activity in MDD. Among patients with MDD to healthy controls (HCs), PET, or fMRI revealed hyperactivity of the amygdala, insula, subcallosal cingulate cortex, and hypoactivity of the dorsolateral prefrontal cortex (Bohning et al., 2001; Zobel et al., 2005; Dunlop et al., 2017). However, heterogeneity in the results was observed among studies, with some patients not showing these changes or even opposite patterns (e.g., PET revealed increased metabolism in the dorsolateral prefrontal cortex) (Goldapple et al., 2004; Dunlop et al., 2017). Furthermore, the published meta-analyses involved either variable patients with recurrent MDD, post-treatment MDD or bipolar MDD, or the region of interest analysis explored, which could not reflect intrinsic whole brain activity. Therefore, there is an urgent need for a meta-analysis of resting-state neuroimaging studies that characterize the brain states of medication-naive patients with first-episode unipolar MDD and potentially provide reliable biomarkers.

In the present study, we used the voxel-based meta-analytic technique anisotropic effect size seed-based d mapping (formerly “signed differential mapping”), which is a voxel-based statistical technique for meta-analyzing studies on differences in brain structure or activity, to perform a whole-brain meta-analysis comparing functional alterations between first-episode medication-naive unipolar MDD patients and HCs. In addition, subgroup meta-analysis was used to control for the MRI analysis method. Moreover, the meta-regression analyses were used to examine the potential effects of mean age, education duration, illness duration and severity of depressive symptoms. Therefore, this meta-analysis may reflect intrinsic brain activity, without the influence of treatment and external tasks, and may provide more reliable information to understand the pathological underpinnings of MDD.

## Methods

### Search Strategy

A systematic search strategy was used to select relevant studies published in PubMed, Ovid and Web of Knowledge between January 1984 and July 2016. The following search terms were used: “ReHo” < or > “regional homogeneity” < or > “ALFF” < or > “amplitude of low frequency fluctuations” < or > “low frequency fluctuations” < or > “ASL” < or > “arterial spin labeling” < or > “CBF” < or > “cerebral blood flow” < or > “rCMRglu” < or > “regional cerebral metabolic” < or > “PET” < or > “positron emission tomography” < or > “SPECT” < or > “single photon emission computed tomography” < or > “neuroimaging”; “depression” < or > “unipolar depression” < or > “depressive disorder” < or > “major depression” < or > “major depressive disorder” < or > “depressed”; and “resting-state” < or> “rest” < or> “resting.”

### Studies Selection

Studies were selected accorded to the following criteria: (1) the original paper used at least one of the functional imaging techniques of fMRI, ASL, PET, or SPECT to analyze whole brain-altered activity in patients with MDD; (2) the study enrolled first-episode medication-naive unipolar MDD patients and a matched HCs group aged 16–60 years; and (3) the article clearly reported 3-dimensional coordinates in the stereotactic space of the activation areas. Studies reporting only region of interest (ROI) findings, the same samples from a previous study, overlapping samples without the largest group, the samples with comorbidity of other psychiatric disorder were excluded. The studies were also excluded if the corresponding authors did not respond to letters ensuring whether the criteria were met or if the data exhibited significant heterogeneity (*p* < 0.005) when added in.

Two authors (XM and TL) independently searched, selected, and cross-checked the literature. The authors discussed any inconsistent articles, reached a consensus decision and implemented the following steps.

### Quality Assessment

The quality of each selected article was independently assessed by two authors using a 10-point checklist based on previous meta-analysis studies (Wang T. et al., 2016; Zhang et al., 2016). The assessment included the quality of the diagnostic procedures, the demographic and clinical characterization, the sample size, the analysis method and the quality of the reported results (see Table S1). Although the checklist was not designed as an assessment tool, it can provide some objective indication of the rigor of individual studies. The study quality scores are presented in Table 1.

**Table 1:** Demographic and clinical characteristics of the participants in 12 fMRI studies (8 ALFF data sets and 4 ReHo data sets) included in the meta-analysis.

**Study**	**Subjects (females**, ***n*****)**		**Age (years)**		**Education (years)**		**Illness duration (months)**	**Severity (scale type)**	**Modality/Analysis**	**Statistical threshold**	**Quality score (out of 10)**
	**MDD**	**HC**	**MDD**	**HC**	**MDD**	**HC**					
Wang L. et al., 2016	35 (23)	32 (20)	33.60	33.70	12.60	13.20	5.10	27.10 (HDRS)	rs-fMRI/ALFF	Corrected	9.5
Du et al., 2016	18 (13)	18 (8)	39.28	35.33	11.63	12.83	–	–	rs-fMRI/ALFF	Corrected	10
Lai and Wu, 2015	44 (23)	27 (15)	36.91	38.29	15.70	15.92	4.68	22.07 (HDRS)	rs-fMRI/ALFF	Uncorrected	10
(Wang L. J. et al., 2014) (END)	30 (17)	33 (19)	35.70	31.45	12.50	12.41	5.48	24.97 (HAMD)	rs-fMRI/ALFF	Corrected	10
(Wang L. J. et al., 2014) (ERD)	26 (16)	33 (19)	32.54	31.45	11.05	12.41	4.00	27.50 (HAMD)	rs-fMRI/ALFF	Corrected	10
Zhang et al., 2014	32 (18)	35 (17)	20.53	20.97	13.88	13.97	–	–	rs-fMRI/ALFF	Corrected	9.5
Liu et al., 2014	30 (17)	30 (15)	29.80	30.10	13.10	14.30	13.3	28.5 (HDRS)	rs-fMRI/ALFF	Corrected	9.5
Shen et al., 2014	16 (9)	14 (8)	34.44	32.36	–	–	2.63	30.88 (HAMD)	rs-fMRI/ALFF	Corrected	9.5
Wang L. et al., 2014	14 (5)	14 (5)	32.93	34.14	13.00	12.97	6.12	26.07 (HAMD)	rs-fMRI/ReHo	Corrected	9.5
Liu et al., 2013	22 (10)	19 (9)	28.09	24.37	12.23	13.11	2.95	25.89 (HDRS)	rs-fMRI/ALFF	Corrected	10
Chen J. D. et al., 2012	15 (6)	15 (7)	24.07	23.93	10.07	12.53	3.3	22.86 (HDRS)	rs-fMRI/ReHo	Corrected	10
Peng et al., 2011	16 (10)	16 (10)	34.1	33.70	14.20	13.50	3.1	31.71 (HDRS)	rs-fMRI/ReHo	Corrected	9.5
Liu et al., 2010	15 (7)	15 (7)	29.13	30.20	12.47	13.47	13.2	32.6 (HAMD)	rs-fMRI/ReHo	Uncorrected	9.5

*HDRS/HAMD, Hamilton Depression Rating Scale*.

### Voxel-Wise Meta-Analysis

A voxel-based meta-analysis was performed to compare resting-state functional activity in first-episode medication-naive unipolar MDD patients and HCs. The meta-analysis was conducted using anisotropic effect size-signed differential mapping (AES-SDM, http://www.sdmproject.com/software) to account for both positive and negative differences between patients and controls, such as hyperactivation and hypoactivation between first-episode medication-naive unipolar MDD patients and HCs, which is a reliable and valid peak coordinate-based method combining both peak coordinates and statistical parametric maps and using standard effect size and variance-based meta-analytic calculations (Radua et al., 2012a, 2014). The three-dimensional peak coordinates normalized to the Montreal Neurological Institute (MNI) space and effect size in each study were extracted for the meta-analysis according to the AES-SDM tutorial. Specifically, the *z*-scores in some studies were converted to t statistics using an online converter (http://www.sdmproject.com/utilities/?show=Statistics). If there was no effect size (*t*-values, *z*-values, *p*-values or similar), then a “*p*” was recorded for positive peaks, and an “*n*” was recorded for negative peaks. AES-SDM set the full width at half maximum (FWHM) to 20 mm because this setting is optimal to balance sensitivity and specificity (Radua et al., 2012a) and may account for some spatial errors, such as cluster size (the larger cluster, the more error between the peak and the center of the cluster) (Ferreira and Busatto, 2010). Other parameters included voxel *P* = 0.005, peak height *Z* = 1, and cluster extent = 10 voxels (Radua et al., 2012a).

### Reliability Analysis

The robustness of the results of the meta-analysis was evaluated through an inspection of heterogeneity, jack-knife and subgroup analyses (Radua et al., 2012b). The inspection of heterogeneity produced a map of the inter-study heterogeneity showing which brain regions are more heterogeneous. The jack-knife sensitivity analysis involved repeating the meta-analysis after excluding one study at a time, and this analysis was used to assess the reproducibility of the results (Radua et al., 2012a). Finally, subgroup meta-analysis was performed to control for the MRI analysis method. If the brain regions are still significant in all or most of the combinative and previous studies, then the findings are highly replicable (Radua et al., 2012a).

### Meta-Regression Analysis

A simple linear regression was used to examine the following variables: mean age, education duration, illness duration, and severity of depressive symptoms. To minimize the detection of spurious associations, we decreased the probability threshold to 0.0005, detected required abnormalities in both the slope and one of the extremes of the regressor, and discarded findings in regions other than those detected in the main analysis, as in previous meta-analyses (Radua et al., 2012a,b). Finally, regression plots were visually inspected to discard fits driven by too few studies (Radua et al., 2012b).

## Results

### Studies Included in the Meta-Analyses

**Figure 1:**
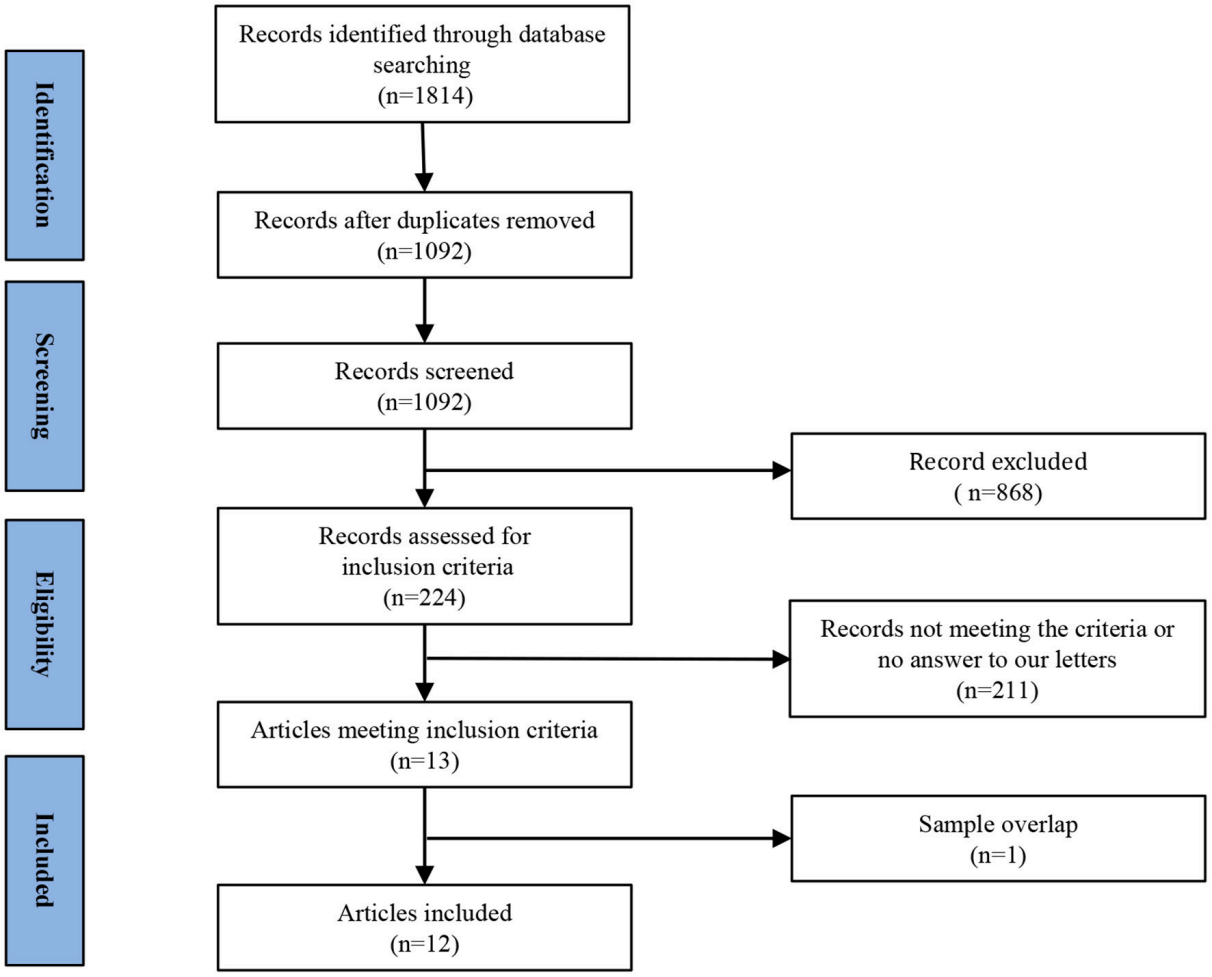
Meta-analysis of resting-state functional activity studies in medication-naive patients with first-episode unipolar major depression compared with healthy controls.

Figure 1 shows a flow diagram of the study selection process. 12 papers (Liu et al., 2010, 2013, 2014; Peng et al., 2011; Chen J. D. et al., 2012; Shen et al., 2014; Wang L. et al., 2014, 2016; Wang L. J. et al., 2014; Zhang et al., 2014; Lai and Wu, 2015; Du et al., 2016) were selected. Specifically, this meta-analysis involved 313 medication-naive patients with first-episode unipolar MDD (55.6% females; mean age 31.62 years) from China, matched with 283 HCs (51.9% females; mean age 30.77 years) based on age, sex, education and nationality. All included studies involved pairwise comparisons between patients and well-matched HCs. Table 1 summarizes the demographic information, clinical data and imaging-specific methodology from all included studies. One study (Wang L. J. et al., 2014) included early treatment-nonresponsive (END) and early treatment-responsive (ERD) patients compared with HCs stratified into Wang et al. (END) and Wang et al. (ERD). Another study (Chen J. D. et al., 2012) extracted partial data involving early-onset depression patients vs. HCs.

### Voxel-Wise Meta-Analysis

**Figure 2:**
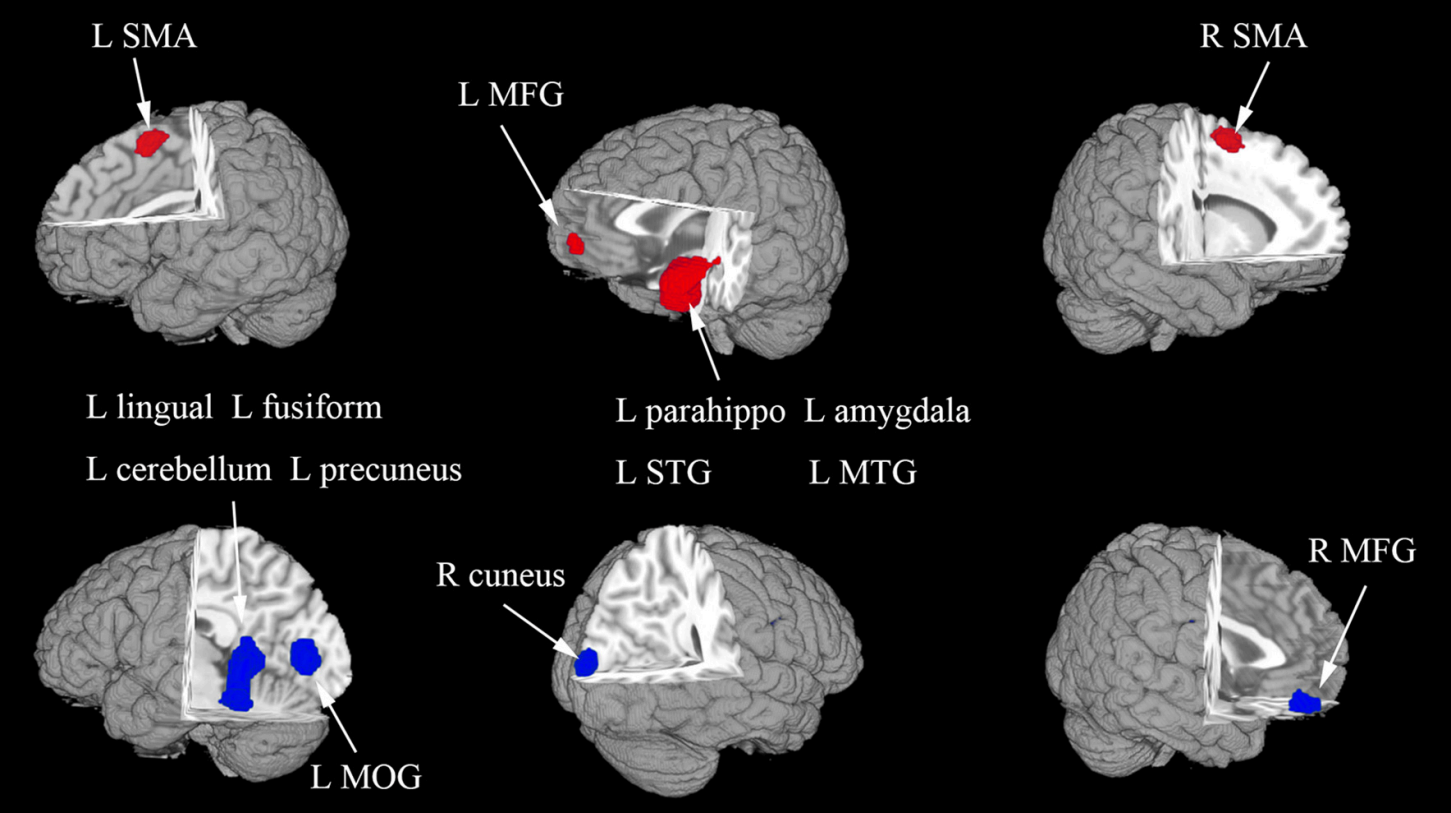
The areas of increased (red) and decreased (blue) resting-state functional activity in the pooled meta-analysis of studies in medication-naive patients with first-episode unipolar major depression compared with healthy controls. R, right; L, left; SMA, supplementary motor area; MFG, middle frontal gyrus; MOG, middle occipital gyrus; STG, superior temporal gyrus; MTG, middle temporal gyrus.

**Figure 3:**
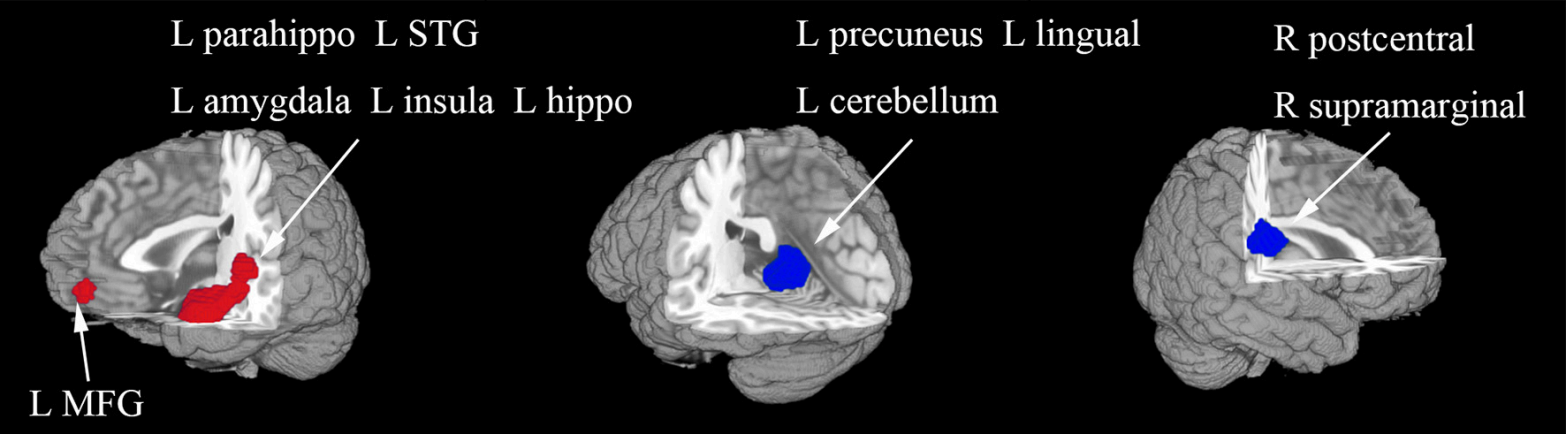
The areas of increased (red) and decreased (blue) resting-state functional activity in the subgroup meta-analysis of “ALFF” studies in medication-naive patients with first-episode unipolar major depression compared with healthy controls. R, right; L, left; STG, superior temporal gyrus; MFG, middle frontal gyrus.

In the whole-brain meta-analysis, first-episode medication-naive unipolar MDD patients showed functional abnormalities compared with HCs. The resting-state functional activity was increased in the left parahippocampal gyrus (PHG, BA 28), left supplementary motor area (SMA; BA 6), and left middle frontal gyrus (MFG; orbital part, BA 11), and decreased in left lingual gyrus (LING; BA 27), left middle occipital gyrus (MOG; BA 19), right cuneus cortex (CUN; BA 18), right MFG (orbital part, BA 11), right supramarginal gyrus (SMAR; BA 48), right postcentral gyrus (PoCG; BA 43), and left cerebellum (including cerebellar hemispheric lobule III/IV/V; BA 30) (Figure 2 and Table 2). In addition, the increased activation in a cluster with peak activation in the left PHG that also extended into the hippocampus and amygdala. The results overlapped in the subgroup meta-analysis of “ALFF” studies. In the subgroup meta-analysis of “ALFF” studies, the resting-state functional activity was increased in both left PHG (BA 28) and left MFG (orbital part, BA 11), and decreased in both left precuneus (BA 30) and right SMAR (BA 48) (Figure 3 and Table S2).

**Table 2:** Brain regions showing greater and less activity in MDD vs. HC (voxel-wise *p* < 0.005 and FWHM = 20 mm).

**Brain regions**	**Maximum**			**Clusters**	
	**Coordinates (MNI) x, y, z**	**SDM value**	***p*-value**	**No. voxel**	**Breakdown(no. of voxels)**
**POOLED META-ANALYSIS**					
**MDD>HC**					
Left parahippocampal gyrus, BA 28	−34, −6, −18	1.770	0.000289023	823	Left parahippocampal gyrus, BA 28, BA 34, BA 35, BA 36 (264)
					Left amygdala, BA20, BA 28, BA 34, BA 36, BA 38, BA 48 (194)
					Left temporal pole, superior temporal gyrus, BA20, BA28, BA34, BA36, BA38, BA 48 (190)
					Left temporal pole, middle temporal gyrus BA 20, BA 35, BA 26 (38)
					Left hippocampus, BA 28, BA 34, BA 35, BA 36 (62)
					Left fusiform gyrus, BA 20, BA 35, BA 36 (42)
					Left olfactory cortex, BA 34, BA 48 (13)
					Left inferior frontal gyrus, orbital part, BA 28, BA 38, BA 34, BA 48 (10)
					Left insula, BA 38, BA 48 (10)
Left supplementary motor area, BA 6	−6, 4, 62	1.565	0.001331508	183	Left supplementary motor area, BA 6 (152)
					Right supplementary motor area, BA 6 (31)
Left middle frontal gyrus, orbital part, BA 11	−22, 60, −10	1.500	0.001992047	41	Left middle frontal gyrus, orbital part, BA 11 (26)
					Left superior frontal gyrus, orbital part, BA 11 (15)
**MDD < HC**					
Left lingual gyrus, BA 27	−12, −44, 0	−1.930	0.000129044	590	Left cerebellum, hemispheric lobule IV / V, BA 18, BA 19, BA 27, BA 30, BA 37 (183)
					Left lingual gyrus, BA 17, BA 18, BA 19, BA 27, BA 30 (146)
					Left fusiform gyrus, BA 30, BA 37 (96)
					Left precuneus, BA 19, BA 27, BA 29, BA 30 (64)
					Left calcarine fissure/surrounding cortex, BA 17, BA 27, BA 29, BA 30 (57)
					Cerebellum, vermic lobule IV / V, BA 27 (30)
					Left posterior cingulate gyrus, BA 29 (8)
					Left parahippocampal gyrus, BA 30 (4)
					Left cerebellum, hemispheric lobule III, BA 30 (2)
Left middle occipital gyrus, BA 19	−42, −78, 8	−1.688	0.000836074	210	Left middle occipital gyrus, BA 19, BA 37, BA 39 (189)
Right cuneus cortex, BA 18	14, −92, 16	−1.631	0.001186967	98	Right cuneus cortex, BA 17, BA 18 (58)
					Right superior occipital gyrus, BA 17, BA 18 (32)
					Right calcarine fissure / surrounding cortex, BA 17, BA 18 (8)
Right middle frontal gyrus, orbital part, BA 11	30, 44, −18	−1.512	0.002404928	72	Right middle frontal gyrus, orbital part, BA 11, BA 47 (69)
					Right inferior frontal gyrus, orbital part, BA 11 (3)
Right supramarginal gyrus, BA 48	60, −18, 22	−1.446	0.003437102	19	Right supramarginal gyrus, BA 43, BA 48 (10)
					Right postcentral gyrus, BA 43, BA 48, (9)
Right postcentral gyrus, BA 43	60, −10, 30	−1.418	0.003906727	16	Right postcentral gyrus, BA 3, BA 43 (16)

*L, Left; R, right; BA, Brodmann area; MNI, Montreal Neurological Institute; M, Middle; SDM, seed-based d mapping*.

### Reliability Analysis

In a whole-brain jack-knife sensitivity analysis of first-episode medication-naive unipolar MDD patients compared with HCs (Table 3), increased functional activity in the left PHG of MDD patients was significant in all. And increased functional activity in the left SMA of MDD patients was also significant in all but 1 combination of the datasets. Increased functional activity in the left MFG (orbital part) and decreased functional activity in the left MOG and right MFG (orbital part) were significant in all but 2 combinations. Decreased functional activity in the left LING remained significant in all but 3 combinations.

**Table 3:** Sensitivity analyses of studies in the meta-analysis of MDD vs. HC.

**Discarded Study**	**Hyperactivation regions**			**Hypoactivation regions**					
	**L PHG**	**L SMA**	**L MFG**	**L LING**	**L MOG**	**R CUN cortex**	**R SMAR**	**R PoCG**	**R MFG**
Wang L. et al., 2016	Y	N	Y	Y	Y	N	Y	N	Y
Du et al., 2016	Y	Y	Y	Y	Y	Y	Y	Y	Y
Lai and Wu, 2015	Y	Y	Y	Y	Y	N	N	N	Y
(Wang L. J. et al., 2014) (END)	Y	Y	N	N	Y	N	N	N	Y
(Wang L. J. et al., 2014) (ERD)	Y	Y	Y	N	Y	N	Y	N	Y
Zhang et al., 2014	Y	Y	Y	Y	Y	N	N	N	N
Liu et al., 2014	Y	Y	N	Y	N	N	N	N	Y
Shen et al., 2014	Y	Y	Y	Y	N	Y	Y	N	Y
Wang L. et al., 2014	Y	Y	Y	Y	Y	N	Y	N	Y
Liu et al., 2013	Y	Y	Y	Y	Y	Y	Y	N	N
Chen J. D. et al., 2012	Y	Y	Y	N	Y	Y	Y	N	Y
Peng et al., 2011	Y	Y	Y	Y	Y	Y	Y	N	Y
Liu et al., 2010	Y	Y	Y	Y	Y	Y	N	Y	Y

*L, left; R, right; PHG, parahippocampal gyrus; SMA, supplementary motor area; MFG, middle frontal gyrus; LING, lingual gyrus; MOG, middle occipital gyrus; CUN, cuneus; SMAR, supramarginal gyrus; PoCG, postcentral gyrus; Y, yes, N, no*.

### Meta-Regression Analysis

The variables explored by regression analyses included mean age, education duration, illness duration and severity of depressive symptoms. The mean illness duration was positively associated with resting-state hyperactivation in the left PHG (BA 28) and hypoactivation in the hemispheric lobule IV/V of left cerebellum (BA 30); no linear association with mean age, education duration and illness severity was observed.

## Discussion

We integrated the findings from 12 resting-state functional neuroimaging studies using AES-SDM, in which first-episode medication-naive unipolar MDD patients were compared with HCs. Without the influence of treatment and external tasks, this whole-brain meta-analysis, comparing functional alterations between first-episode medication-naive unipolar MDD patients and HCs, could reflect intrinsic brain activity and may provide more reliable information on the neural patterns in some regions and their potential roles in the pathophysiology of MDD, which is different from previous meta-analyses. The results of the present meta-analysis are largely consistent with previous MDD studies, showing a subset of regional differences, including hyperactive left PHG, left SMA, left MFG (orbital part), and hypoactive left LING, left MOG, right CUN cortex, right MFG (orbital part), right SMAR, and right PoCG. In addition, differences were observed in other regions, such as hyperactive left hippocampus, which have hitherto been neglected in studies of first-episode medication-naive unipolar MDD patients. The results of subgroup meta-analysis of “ALFF” were similar. Therefore, these results demonstrated the reproducibility and comparability of these studies.

### Findings in the Pooled Meta-Analysis

In the pooled meta-analysis, MDD patients showed increased resting-state brain activity in the left PHG, left amygdala, and left hippocampus, and decreased activity in the left cerebellum (including cerebellar hemispheric lobule III/IV/V).

Over the last decade, a neurobiological limbic-cortical model of MDD has been described. Using this model, Mayberg (2003) and Mayberg et al. (1999) suggested that depression is related to hyperactivity in limbic areas associated with emotional processing and inadequate inhibition by prefrontal areas. The hyperactive regions include the amygdala, hippocampus and orbitofrontal cortex, consistent with the results of the present study.

The amygdala is a key structure in a limbic circuit and plays an important role in involuntary, automatic appraisal processes, which are crucial components of emotion (Dannlowski et al., 2007). The amygdala is likely involved in the pathophysiology of depression, because several studies have used various imaging modalities, such as structural MRI (Lorenzetti et al., 2010; Burke et al., 2011), task-related MRI (Thomas et al., 2001) and rs-fMRI (Du et al., 2016), to report amygdala alterations in patients with depression. Previous meta-analyses (Campbell et al., 2004; Arnone et al., 2012) have reported the lack of volume differences in the amygdala between patients with depression and HCs. However, the results do not exclude the involvement of amygdala in depression. Based on facial recognition tasks, fMRI studies have reported abnormal BOLD signals in the amygdala, showing hyperactivation or hypoactivation compared with controls or in response to treatment (Thomas et al., 2001; Fu et al., 2004; Anand et al., 2007). The role of the left amygdala in depression is not only supported by emotion task studies but also by rs-fMRI studies. Du et al. (2016) showed that ALFF increased in the bilateral amygdala, and this study was included in the present meta-analysis. However, we observed hyperactivity in the amygdala on the left side. This finding may reflect the heterogeneity of the right amygdala in MDD. It is also likely that the hyperactivity in the right amygdala may also reflect differential responses to the confining environment, such as the MRI scanner, and different characteristics of subjects. Further studies are needed to determine their relative contributions to MDD pathology. A previous study (Mandell et al., 2014; Palmer et al., 2014) illustrated that the core role of increased activity in the amygdala is consistent with rumination hypotheses, and the hyperactive amygdala may be associated with the identification and processing of negative stimuli, guiding attention toward potential danger (Davis and Whalen, 2001). The results of the present study suggest that left hyperactive amygdala in depression affects the onset and maintenance of emotional disorders by eliciting dysfunctional negative biases at automatic stages of affective information processing (Dannlowski et al., 2007).

The hippocampus is also likely involved in the pathophysiology of depression. Some studies have proposed several putative genetic risk variants associated with hippocampal structure (Baune et al., 2012; Dannlowski et al., 2015). Previous meta-analyses have reported a reduction in hippocampal size in MDD patients, associated with the illness duration in untreated depression patients (Sheline et al., 2003), and suggested its possible use as a diagnostic neuro-biomarker for MDD (Cole et al., 2011; Wang et al., 2012; Kambeitz et al., 2016). The reduced hippocampal volume may be accompanied by altered function, which has been neglected in studies of first-episode medication-naive unipolar MDD patients. In the present study, we detected an increase in left hippocampal activity, consistent with other studies (Milne et al., 2012). The hippocampus plays a role in assessing novel items, information retrieval success, visual memory, spatial memory, and recollection memory (Tulving et al., 1994; Nyberg et al., 1996; Yonelinas, 1997; Bellgowan et al., 2003; Rugg and Yonelinas, 2003; Diana et al., 2007; Milne et al., 2012). Hippocampal activation was most consistent with proposed memory models correlating the hippocampus with recollection memory (Diana et al., 2007). The results of the present study suggest that the left hippocampus, with reduced volume and hyperactivity in depression, could serve as a neuroimaging biomarker for diagnosing MDD.

The anterior cerebellum is also likely involved in the pathophysiology of depression. Several MDD studies (Liu et al., 2010, 2014) have identified abnormal cerebellar activity and these results are largely consistent with the findings of the present study, showing left anterior cerebellar hypoactivity in MDD. Although findings in the cerebellum are uncommon in early neuroimaging studies, these studies have illustrated growing evidence that the cerebellum, long considered to possess a sensorimotor function, plays a role in the cognitive and emotional processing of negative stimuli (Schraa-Tam et al., 2012; Wang et al., 2012). Patients with cerebellar lesions show a range of emotional and behavioral abnormalities, including distractibility, disinhibition, anxiety and irritability (Schmahmann et al., 2007). The literature has reported that D-amino acid oxidase activator gene is associated with a hypoactive cerebellum in patients with depression, suggesting that glutamatergic modulation will influence the cerebellar activity in MDD (Chen J. et al., 2012; Lai and Wu, 2016). Liu et al. (2012) reported increased ReHo values in the cerebellum in MDD patients. Discrepancies among the observations in the cerebellum likely reflect different characteristics of subjects, such as age, the MRI scanner used and analytic modifications. Furthermore, a resting-state functional connectivity study showed that decreased functional connectivity between the left cerebellum and inferior parietal lobule may be a disease state phenomenon (Liu et al., 2013) and represent the characteristics of adult first-episode medication-naive unipolar MDD patients (Lai and Wu, 2016). Taken together, these findings suggest that the left hypoactive anterior cerebellum at rest may contribute to internal activity in MDD.

### Findings in the Subgroup Meta-Analysis

The subgroup meta-analysis of “ALFF” studies revealed hyperactivity in the left PHG, left amygdala, left hippocampus, and left rolandic operculum in MDD compared with HCs. In particular, hyperactive left rolandic operculum was not identified in the pooled meta-analysis. To our knowledge, this study is the first to report this finding. Tozzi et al. (2016) observed increased mean diffusivity (MD) and fractional anisotropy (FA) values in the left rolandic operculum in patients carrying the T allele of rs1360780 of the FKBP5 gene, which has been associated with depression (Gillespie et al., 2009; Lavebratt et al., 2010) and white-matter abnormalities (Fani et al., 2014), compared with patients homozygous for the C allele. Increased MD and reduced FA are associated with axonal degeneration, demyelination, decreased axonal density, and incomplete white-matter maturation (Feldman et al., 2010; Alexander et al., 2011). These structural changes may be associated with the altered function observed in the current subgroup meta-analysis of “ALFF.” Thus, further studies are warranted.

### Findings in the Meta-Regression Analysis

Meta-regression analyses of MDD vs. HC studies showed that the mean illness duration was positively associated with resting-state hyperactivation in the left PHG. To our knowledge, this study is the first to report this positive relationship. The PHG is a limbic circuit structure associated with mood regulation. The left hyperactive PHG may lead to a disconnection syndrome, which partly contributes to emotional dysregulation exhibited by patients with MDD (Liu et al., 2013). Previous studies (Chen J. D. et al., 2012; Liu et al., 2013) have reported increased activity in the left hyperactive PHG, consistent with the pooled meta-analysis in the present study. However, other researches have shown decreased areas in a study of patients with long depression histories (Radua et al., 2012b). Thus, we suggest that the increased left PHG activity might accompany the development of MDD. Further studies are warranted.

We also observed that the mean illness duration was positively associated with resting-state hypoactivation in the hemispheric lobule IV/V of the left cerebellum, consistent with a previous study (Wang L. J. et al., 2014). Notably, we did not detect a linear association with mean age, education duration and illness severity.

### Limitations

The present meta-analysis has some limitations. First, the availability of studies meeting the criteria for selection may limit the power of our analyses. The exclusion of studies that used functional connectivity approaches or did not report stereotaxic coordinates likely reduced the power to detect less-robust activations. Second, the small number of studies precluded separate meta-analyses for some moderator variables, such as the characteristics of patients (gender). Although we conducted subgroup meta-analysis of “ALFF” in MDD compared with HCs, this analysis included only 8 studies and had limited power; further studies are needed. Third, the accuracy of this voxel-wise meta-analysis may have been limited, because AES-SDM, like all coordinate-based methods, assumes that effect sizes originate from homogeneous *t*-value contrasts; in fact, these effects might originate from different covariate models or from different raw statistics, and this limitation could be controlled using SDM covariate analyses where relevant (Wang T. et al., 2016). Finally, all neuroimaging data are highly sensitive to common artifacts, such as breathing effect and head motion, which may influence the results.

## Conclusions

The present voxel-wise meta-analysis provided a unique opportunity to assess altered resting-state brain activity across individual MDD studies. The results confirmed a subset of regional differences reported in previous MDD studies, including hyperactivity in the left parahippocampal gyrus, left supplementary motor area, left amygdala and left MFG (orbital part), and hypoactivity in the left LING, left MOG, right CUN, right MFG (orbital part), and left cerebellum (including cerebellar hemispheric lobule III/IV/V). Additional regions were observed, such as left hippocampus, left rolandic operculum, left PHG, which had received less attention. Further studies are needed to determine whether the findings reported here are disease-related. The innovative methodological approach studies that transcend limitations mentioned above and longitudinal studies that investigate the dynamic brain activity changes of MDD patients and the relationship between these alterations and cognition will help us better understand the neuropathological changes in MDD patients.

## Author Contributions

All authors provided substantial contributions to the work. XM, JL, QG, and MW conceived and designed the study. XM, JL, TL, LM, WW, SS, and YW analyzed the data and performed the statistical study. XM drafted the manuscript. JL and MW revised the manuscript. After revisions and editing by all authors, the article was submitted.

### Conflict of Interest Statement

The authors declare that the research was conducted in the absence of any commercial or financial relationships that could be construed as a potential conflict of interest.

## References

[B1] Alexander A. L. Hurley S. A. Samsonov A. A. Adluru N. Hosseinbor A. P. Mossahebi P.. (2011). Characterization of cerebral white matter properties using quantitative magnetic resonance imaging stains. Brain Connect. 1, 423–446. 10.1089/brain.2011.0071 22432902 PMC3360545

[B2] Anand A. Li Y. Wang Y. Gardner K. Lowe M. J. (2007). Reciprocal effects of antidepressant treatment on activity and connectivity of the mood regulating circuit: an FMRI study. J. Neuropsychiatr. Clin. Neurosci. 19, 274–282. 10.1176/jnp.2007.19.3.274 17827412 PMC3465666

[B3] Arnone D. McIntosh A. M. Ebmeier K. P. Munafo M. R. Anderson I. M. (2012). Magnetic resonance imaging studies in unipolar depression: systematic review and meta-regression analyses. Eur. Neuropsychopharmacol. 22, 1–16. 10.1016/j.euroneuro.2011.05.003 21723712

[B4] Baune B. T. Konrad C. Grotegerd D. Suslow T. Birosova E. Ohrmann P.. (2012). Interleukin-6 gene (IL-6): a possible role in brain morphology in the healthy adult brain. J. Neuroinflammation 9:125. 10.1186/1742-2094-9-125 22695063 PMC3464888

[B5] Bellgowan P. S. Saad Z. S. Bandettini P. A. (2003). Understanding neural system dynamics through task modulation and measurement of functional MRI amplitude, latency, and width. Proc. Natl. Acad. Sci. U S A. 100, 1415–1419. 10.1073/pnas.0337747100 12552093 PMC298787

[B6] Bohning D. E. Lomarev M. P. Denslow S. Nahas Z. Shastri A. George M. S. (2001). Feasibility of vagus nerve stimulation-synchronized blood oxygenation leveldependent functional MRI. Invest. Radiol. 36, 470–479. 10.1097/00004424-200108000-00006 11500598

[B7] Burke J. McQuoid D. R. Payne M. E. Steffens D. C. Krishnan R. R. Taylor W. D. (2011). Amygdala volume in late-life depression: relationship with age of onset. Am. J. Geriatr. Psychiatry 19, 771–776. 10.1097/JGP.0b013e318211069a 21873832 PMC3164525

[B8] Caan W. (2015). The global crisis of depression: the low of the 21st century? Perspect. Public Health 135:62. 10.1177/1757913915569958 25759310

[B9] Campbell S. Marriott M. Nahmias C. MacQueen G. M. (2004). Lower hippocampal volume in patients suffering from depression: a meta-analysis. Am. J. Psychiatry 161, 598–607. 10.1176/appi.ajp.161.4.598 15056502

[B10] Chen J. Xu Y. Zhang J. Liu Z. Xu C. Zhang K.. (2012). Genotypic association of the DAOA gene with resting-state brain activity in major depression. Mol. Neurobiol. 46, 361–373. 10.1007/s12035-012-8294-5 22851402

[B11] Chen J. D. Liu F. Xun G. L. Chen H. F. Hu M. R. Guo X. F.. (2012). Early and late onset, first-episode, treatment-naive depression: same clinical symptoms, different regional neural activities. J. Affect. Disord. 143, 56–63. 10.1016/j.jad.2012.05.025 22749158

[B12] Cole J. Costafreda S. G. McGuffin P. Fu C. H. (2011). Hippocampal atrophy in first episode depression: a meta-analysis of magnetic resonance imaging studies. J. Affect. Disord. 134, 483–487. 10.1016/j.jad.2011.05.057 21745692

[B13] Dannlowski U. Kugel H. Grotegerd D. Redlich R. Suchy J. Opel N.. (2015). NCAN cross-disorder risk variant is associated with limbic gray matter deficits in healthy subjects and major depression. Neuropsychopharmacology 40, 2510–2516. 10.1038/npp.2015.86 25801500 PMC4569958

[B14] Dannlowski U. Ohrmann P. Bauer J. Kugel H. Arolt V. Heindel W.. (2007). Amygdala reactivity predicts automatic negative evaluations for facial emotions. Psychiatry Res. 154, 13–20. 10.1016/j.pscychresns.2006.05.005 17182226

[B15] Davis M. Whalen P. J. (2001). The amygdala: vigilance and emotion. Mol. Psychiatry 6, 13–34. 10.1038/sj.mp.4000812 11244481

[B16] Diana R. A. Yonelinas A. P. Ranganath C. (2007). Imaging recollection and familiarity in the medial temporal lobe: a three-component model. Trends Cogn. Sci. 11, 379–386. 10.1016/j.tics.2007.08.001 17707683

[B17] Du L. Wang J. Meng B. Yong N. Yang X. Huang Q.. (2016). Early life stress affects limited regional brain activity in depression. Sci. Rep. 6:25338. 10.1038/srep25338 27138376 PMC4853783

[B18] Dunlop B. W. Rajendra J. K. Craighead W. E. Kelley M. E. McGrath C. L. Choi K. S. (2017). Functional connectivity of the subcallosal cingulate cortex and differential outcomes to treatment with cognitive-behavioral therapy or antidepressant medication for major depressive disorder. Am. J. Psychiatry 174, 533–545. 10.1176/appi.ajp.2016.16050518 28335622 PMC5453828

[B19] Fani N. King T. Z. Reiser E. Binder E. B. Jovanovic T. Bradley B.. (2014). FKBP5 genotype and structural integrity of the posterior cingulum. Neuropsychopharmacology 39, 1206–1213. 10.1038/npp.2013.322 24253961 PMC3957115

[B20] Feldman H. M. Yeatman J. D. Lee E. S. Barde L. H. Gaman-Bean S. (2010). Diffusion tensor imaging: a review for pediatric researchers and clinicians. J. Dev. Behav. Pediatr. 31, 346–356. 10.1097/DBP.0b013e3181dcaa8b 20453582 PMC4245082

[B21] Ferreira L. K. Busatto G. F. (2010). Heterogeneity of coordinate-based meta-analyses of neuroimaging data: an example from studies in OCD. Br. J. Psychiatry 197, 76–77. 10.1192/bjp.197.1.76a 20592442

[B22] Fried E. I. Nesse R. M. (2015). Depression sum-scores don't add up: why analyzing specific depression symptoms is essential. BMC Med. 13:72. 10.1186/s12916-015-0325-4 25879936 PMC4386095

[B23] Fu C. H. Williams S. C. Cleare A. J. Brammer M. J. Walsh N. D. Kim J.. (2004). Attenuation of the neural response to sad faces in major depression by antidepressant treatment: a prospective, event-related functional magnetic resonance imaging study. Arch. Gen. Psychiatry 61, 877–889. 10.1001/archpsyc.61.9.877 15351766

[B24] Gillespie C. F. Phifer J. Bradley B. Ressler K. J. (2009). Risk and resilience: genetic and environmental influences on development of the stress response. Depress. Anxiety 26, 984–992. 10.1002/da.20605 19750552 PMC2852579

[B25] Goldapple K. Segal Z. Garson C. Lau M. Bieling P. Kennedy S.. (2004). Modulation of cortical-limbic pathways in major depression: treatment-specific effects of cognitive behavior therapy. Arch. Gen. Psychiatry 61, 34–41. 10.1001/archpsyc.61.1.34 14706942

[B26] Kambeitz J. Cabral C. Sacchet M. D. Gotlib I. H. Zahn R. Serpa M. H.. (2016). Detecting neuroimaging biomarkers for depression: a meta-analysis of multivariate pattern recognition studies. Biol. Psychiatry 82, 330–338. 10.1016/j.biopsych.2016.10.028 28110823 PMC11927514

[B27] Lai C. H. Wu Y. T. (2015). The patterns of fractional amplitude of low-frequency fluctuations in depression patients: the dissociation between temporal regions and fronto-parietal regions. J. Affect. Disord. 175, 441–445. 10.1016/j.jad.2015.01.054 25679198

[B28] Lai C. H. Wu Y. T. (2016). The alterations in regional homogeneity of parieto-cingulate and temporo-cerebellum regions of first-episode medication-naive depression patients. Brain Imaging Behav. 10, 187–194. 10.1007/s11682-015-9381-9 25904155

[B29] Lavebratt C. Aberg E. Sjoholm L. K. Forsell Y. (2010). Variations in FKBP5 and BDNF genes are suggestively associated with depression in a Swedish population-based cohort. J. Affect. Disord. 125, 249–255. 10.1016/j.jad.2010.02.113 20226536

[B30] Liu F. Guo W. Liu L. Long Z. Ma C. Xue Z.. (2013). Abnormal amplitude low-frequency oscillations in medication-naive, first-episode patients with major depressive disorder: a resting-state fMRI study. J. Affect. Disord. 146, 401–406. 10.1016/j.jad.2012.10.001 23116810

[B31] Liu F. Hu M. Wang S. Guo W. Zhao J. Li J.. (2012). Abnormal regional spontaneous neural activity in first-episode, treatment-naive patients with late-life depression: a resting-state fMRI study. Prog. Neuropsychopharmacol. Biol. Psychiatry 39, 326–331. 10.1016/j.pnpbp.2012.07.004 22796277

[B32] Liu J. Ren L. Womer F. Y. Wang J. Fan G. Jiang W.. (2014). Alterations in amplitude of low frequency fluctuation in treatment-naive major depressive disorder measured with resting-state fMRI. Hum. Brain Mapp. 35, 4979–4988. 10.1002/hbm.22526 24740815 PMC6869357

[B33] Liu Z. Xu C. Xu Y. Wang Y. Zhao B. Lv Y.. (2010). Decreased regional homogeneity in insula and cerebellum: a resting-state fMRI study in patients with major depression and subjects at high risk for major depression. Psychiatry Res. 182, 211–215. 10.1016/j.pscychresns.2010.03.004 20493670

[B34] Lorenzetti V. Allen N. B. Whittle S. Yucel M. (2010). Amygdala volumes in a sample of current depressed and remitted depressed patients and healthy controls. J. Affect. Disord. 120, 112–119. 10.1016/j.jad.2009.04.021 19464062

[B35] Mandell D. Siegle G. J. Shutt L. Feldmiller J. Thase M. E. (2014). Neural substrates of trait ruminations in depression. J. Abnorm. Psychol. 123, 35–48. 10.1037/a0035834 24661157 PMC4128503

[B36] Mayberg H. S. (2003). Modulating dysfunctional limbic-cortical circuits in depression: towards development of brain-based algorithms for diagnosis and optimised treatment. Br. Med. Bull. 65, 193–207. 10.1093/bmb/65.1.193 12697626

[B37] Mayberg H. S. Liotti M. Brannan S. K. McGinnis S. Mahurin R. K. Jerabek P. A.. (1999). Reciprocal limbic-cortical function and negative mood: converging PET findings in depression and normal sadness. Am. J. Psychiatry 156, 675–682. 10327898 10.1176/ajp.156.5.675

[B38] Milne A. M. MacQueen G. M. Hall G. B. (2012). Abnormal hippocampal activation in patients with extensive history of major depression: an fMRI study. J. Psychiatry Neurosci. 37, 28–36. 10.1503/jpn.110004 21745440 PMC3244496

[B39] Nyberg L. McIntosh A. R. Cabeza R. Habib R. Houle S. Tulving E. (1996). General and specific brain regions involved in encoding and retrieval of events: what, where, and when. Proc. Natl. Acad. Sci. U S A. 93, 11280–11285. 10.1073/pnas.93.20.11280 8855347 PMC38321

[B40] Palmer S. M. Crewther S. G. Carey L. M. Team S. P. (2014). A meta-analysis of changes in brain activity in clinical depression. Front. Hum. Neurosci. 8:1045. 10.3389/fnhum.2014.01045 25642179 PMC4294131

[B41] Peng D. H. Jiang K. D. Fang Y. R. Xu Y. F. Shen T. Long X. Y.. (2011). Decreased regional homogeneity in major depression as revealed by resting-state functional magnetic resonance imaging. Chin. Med. J. 124, 369–373. 10.3760/cma.j.issn.0366-6999.2011.03.009 21362335

[B42] Radua J. Borgwardt S. Crescini A. Mataix-Cols D. Meyer-Lindenberg A. McGuire P. K.. (2012b). Multimodal meta-analysis of structural and functional brain changes in first episode psychosis and the effects of antipsychotic medication. Neurosci. Biobehav. Rev. 36, 2325–2333. 10.1016/j.neubiorev.2012.07.012 22910680

[B43] Radua J. Mataix-Cols D. Phillips M. L. El-Hage W. Kronhaus D. M. Cardoner N.. (2012a). A new meta-analytic method for neuroimaging studies that combines reported peak coordinates and statistical parametric maps. Eur. Psychiatry 27, 605–611. 10.1016/j.eurpsy.2011.04.001 21658917

[B44] Radua J. Rubia K. Canales-Rodriguez E. J. Pomarol-Clotet E. Fusar-Poli P. Mataix-Cols D. (2014). Anisotropic kernels for coordinate-based meta-analyses of neuroimaging studies. Front. Psychiatry 5:13. 10.3389/fpsyt.2014.00013 24575054 PMC3919071

[B45] Rosenstrom T. Jokela M. (2017). Reconsidering the definition of major depression based on collaborative psychiatric epidemiology surveys. J. Affect. Disord. 207, 38–46. 10.1016/j.jad.2016.09.014 27690352

[B46] Rugg M. D. Yonelinas A. P. (2003). Human recognition memory: a cognitive neuroscience perspective. Trends Cogn. Sci. 7, 313–319. 10.1016/S1364-6613(03)00131-1 12860190

[B47] Schmahmann J. D. Weilburg J. B. Sherman J. C. (2007). The neuropsychiatry of the cerebellum - insights from the clinic. Cerebellum 6, 254–267. 10.1080/14734220701490995 17786822

[B48] Schraa-Tam C. K. Rietdijk W. J. Verbeke W. J. Dietvorst R. C. van den Berg W. E. Bagozzi R. P.. (2012). fMRI activities in the emotional cerebellum: a preference for negative stimuli and goal-directed behavior. Cerebellum 11, 233–245. 10.1007/s12311-011-0301-2 21761197 PMC3311856

[B49] Sheline Y. I. Gado M. H. Kraemer H. C. (2003). Untreated depression and hippocampal volume loss. Am. J. Psychiatry 160, 1516–1518. 10.1176/appi.ajp.160.8.1516 12900317

[B50] Shen T. Qiu M. Li C. Zhang J. Wu Z. Wang B.. (2014). Altered spontaneous neural activity in first-episode, unmedicated patients with major depressive disorder. Neuroreport 25, 1302–1307. 10.1097/WNR.0000000000000263 25229945

[B51] Thomas K. M. Drevets W. C. Dahl R. E. Ryan N. D. Birmaher B. Eccard C. H.. (2001). Amygdala response to fearful faces in anxious and depressed children. Arch. Gen. Psychiatry 58, 1057–1063. 10.1001/archpsyc.58.11.1057 11695953

[B52] Tozzi L. Carballedo A. Wetterling F. McCarthy H. O'Keane V. Gill M.. (2016). Single-nucleotide polymorphism of the FKBP5 gene and childhood maltreatment as predictors of structural changes in brain areas involved in emotional processing in depression. Neuropsychopharmacology 41, 487–497. 10.1038/npp.2015.170 26076833 PMC5130124

[B53] Tulving E. Kapur S. Craik F. I. Moscovitch M. Houle S. (1994). Hemispheric encoding/retrieval asymmetry in episodic memory: positron emission tomography findings. Proc. Natl. Acad. Sci. U S A. 91, 2016–2020. 10.1073/pnas.91.6.2016 8134342 PMC43300

[B54] Wang L. Hermens D. F. Hickie I. B. Lagopoulos J. (2012). A systematic review of resting-state functional-MRI studies in major depression. J. Affect. Disord. 142, 6–12. 10.1016/j.jad.2012.04.013 22858266

[B55] Wang L. Kong Q. Li K. Su Y. Zeng Y. Zhang Q.. (2016). Frequency-dependent changes in amplitude of low-frequency oscillations in depression: a resting-state fMRI study. Neurosci. Lett. 614, 105–111. 10.1016/j.neulet.2016.01.012 26797652

[B56] Wang L. Li K. Zhang Q. Zeng Y. Dai W. Su Y.. (2014). Short-term effects of escitalopram on regional brain function in first-episode drug-naive patients with major depressive disorder assessed by resting-state functional magnetic resonance imaging. Psychol. Med. 44, 1417–1426. 10.1017/S0033291713002031 23942213

[B57] Wang L. J. Kuang W. H. Xu J. J. Lei D. Yang Y. C. (2014). Resting-state brain activation correlates with short-time antidepressant treatment outcome in drug-naive patients with major depressive disorder. J. Int. Med. Res. 42, 966–975. 10.1177/0300060514533524 24898399

[B58] Wang T. Liu J. Zhang J. Zhan W. Li L. Wu M.. (2016). Altered resting-state functional activity in posttraumatic stress disorder: a quantitative meta-analysis. Sci. Rep. 6:27131. 10.1038/srep27131 27251865 PMC4890007

[B59] Yonelinas A. P. (1997). Recognition memory ROCs for item and associative information: the contribution of recollection and familiarity. Mem. Cognit. 25, 747–763. 10.3758/BF03211318 9421560

[B60] Zhang H. Li L. Wu M. Chen Z. Hu X. Chen Y.. (2016). Brain gray matter alterations in first episodes of depression: a meta-analysis of whole-brain studies. Neurosci. Biobehav. Rev. 60, 43–50. 10.1016/j.neubiorev.2015.10.011 26592799

[B61] Zhang X. Zhu X. Wang X. Zhu X. Zhong M. Yi J.. (2014). First-episode medication-naive major depressive disorder is associated with altered resting brain function in the affective network. PloS ONE 9:e85241. 10.1371/journal.pone.0085241 24416367 PMC3887023

[B62] Zobel A. Joe A. Freymann N. Clusmann H. Schramm J. Reinhardt M.. (2005). Changes in regional cerebral blood flow by therapeutic vagus nerve stimulation in depression: an exploratory approach. Psychiatry Res. 139,165–179. 10.1016/j.pscychresns.2005.02.010 16043331

