# Supplementary material for: Altered Resting-State Functional Activity in Medication-Naive Patients With First-Episode Major Depression Disorder vs. Healthy Control: A Quantitative Meta-Analysis
Source: Front Behav Neurosci. 2019 May 7;13:89. doi: 10.3389/fnbeh.2019.00089 (PMC6524692; doi:10.3389/fnbeh.2019.00089)
Supplement: Supplementary file 1 [file Table_1.DOC]

**Table S1 Quality Assessment Checklist (When criteria were partially met, 0.5 points were assigned)**

| **Category 1: Participants** | Score (0/0.5/1) |
| --- | --- |
| 1. Patients were evaluated prospectively, specific diagnostic criteria were applied, and demographic data were reported.  2. Comparison participants were evaluated prospectively psychiatric and medical illnesses were excluded.  3. Important variables (e.g., age, sex, illness duration, onset, medication status, BMI, HbA1c, intelligence quotient, i.e. IQ, handedness) were checked either by stratification or statistically.  4. Sample size per group > 10. | |
| **Category 2: Methods for image acquisition and analysis** | |
| 5. Whole brain analysis was automated with no a priori regional selection.  6. Coordinates reported in a standard space.  7. The imaging technique used was clearly described so that it could be reproduced.  8. Measurements were clearly described so that they could be reproduced. | |
| **Category 3: Results and conclusions** | |
| 9. Statistical parameters for significant and important non-significant differences were provided.  10. Conclusions were consistent with the results obtained and the limitations were discussed. | |
| TOTAL /10 | |

**Table S2** Brain regions showing greater and less activity in MDD vs. HC in the subgroup meta-analysis (voxel-wise p < 0.005 and FWHM=20 mm).

|  | Maximum | | |  | Clusters | |
| --- | --- | --- | --- | --- | --- | --- |
| Brain Regions | coordinates  (MNI)  x, y, z | SDM value | p-value |  | No. voxel | Breakdown  (no. of voxels) |
| Subgroup meta-analysis of ALFF | | | | | | |
| MDD > HC | | | | | | |
| Left parahippocampal gyrus, BA 28 | -38, -6, -16 | 2.006 | 0.000180602 | | 990 | Left parahippocampal gyrus, BA 28, BA 34, BA 35, BA 36 (252) |
|  |  |  |  | |  | Left temporal pole, superior temporal gyrus, BA 20, BA 28, BA 34, BA 36, BA 38, BA 48 (201) |
|  |  |  |  | |  | Left amygdala, BA 20 BA 34, BA 36, BA 38, BA 48, (197) |
|  |  |  |  | |  | Left insula, BA 38, BA 48 (102) |
|  |  |  |  | |  | Left hippocampus, BA 28, BA 34, BA 35, BA 36, BA 48 (75) |
|  |  |  |  | |  | Left superior temporal gyrus, BA 48 (73) |
|  |  |  |  | |  | Left olfactory cortex, BA 48, BA 34, (26) |
|  |  |  |  | |  | Left rolandic operculum, BA 48 (20) |
|  |  |  |  | |  | Left heschl gyrus, BA 48 (19) |
|  |  |  |  | |  | Left inferior frontal gyrus, orbital part, BA 38, BA 28, BA 34, (13) |
|  |  |  |  | |  | Left fusiform gyrus, BA 36 (7) |
|  |  |  |  | |  | Left temporal pole, middle temporal gyrus, BA 20, BA 36 (5) |
| Left middle frontal gyrus, orbital part, BA 11 | -22,62, -12 | 1.552 | 0.002053976 | | 65 | Left superior frontal gyrus, orbital part, BA 11 (33) |
|  |  |  |  | |  | Left middle frontal gyrus, orbital part, BA 11 (32) |
| MDD <HC | | | | | | |
| Left precuneus, BA 30 | -4, -52,12 | -1.635 | 0.000464499 | | 370 | Left lingual gyrus, BA 17, BA 18, BA 27, BA 30 (88) |
|  |  |  |  | |  | Left precuneus, BA 17, BA 19, BA 27, BA 29 BA 30 (86) |
|  |  |  |  | |  | Cerebellum, vermic lobule IV / V, BA 27 (68) |
|  |  |  |  | |  | Left calcarine fissure / surrounding cortex, BA 17, BA 27, BA 29, BA 30 (62) |
|  |  |  |  | |  | Left cerebellum, hemispheric lobule IV / V, BA 18, BA 27, BA 30 (54) |
|  |  |  |  | |  | Left posterior cingulate gyrus, BA 29, BA 30 (12) |
| Right supramarginal gyrus, BA 48 | 60, -14,24 | -1.589 | 0.000583172 | | 145 | Right postcentral gyrus, BA 43, BA 48 (95) |
|  |  |  |  | |  | Right supramarginal gyrus, BA 43, BA 48 (45) |
